# Prokaryotic Expression of *α*-13 Giardin Gene and Its Intracellular Localization in* Giardia lamblia*

**DOI:** 10.1155/2017/1603264

**Published:** 2017-02-14

**Authors:** Xingang Yu, Auwalu Yusuf Abdullahi, Sheng Wu, Weida Pan, Xianli Shi, Wei Hu, Liping Tan, Kangxin Li, Zhen Wang, Guoqing Li

**Affiliations:** Guangdong Provincial Zoonosis Prevention and Control Key Laboratory, College of Veterinary Medicine, South China Agricultural University, Guangzhou 510542, China

## Abstract

To study prokaryotic expression and subcellular localization of *α*-13 giardin in* Giardia lamblia* trophozoites, *α*-13 giardin gene was amplified and cloned into prokaryotic expression vector pET-28a(+). The positive recombinant plasmid was transformed into* E. coli* BL21(DE3) for expression by using IPTG and autoinduction expression system (ZYM-5052). The target protein was validated by SDS-PAGE and Western blotting and purified by Ni-NTA Resin. Rabbits were immunized with purified fusion proteins for preparation of polyclonal antibody; then the intracellular location of *α*-13 giardin was determined by fluorescence immunoassay. The results showed that the length of *α*-13 giardin gene was 1038 bp, encoding a polypeptide of 345 amino acids. The expressed product was a fusion protein with about 40 kDa largely present in soluble form. The target protein accounted for 21.0% of total proteins after being induced with IPTG, while it accounted for 28.8% with ZYM-5052. The anti-*α*13-giardin polyclonal antibody possessed good antigenic specificity as well as excellent binding activity with recombinant *α*-13 giardin. Immunofluorescence assays revealed that *α*-13 giardin was localized in the cytoplasm of* G. lamblia* trophozoite, suggesting that it is a cytoplasm-associated protein. The present study may lay a foundation for further functional research on *α*-13 giardin of* G. lamblia*.

## 1. Introduction

*Giardia lamblia* (syn.* G. intestinalis, G. duodenalis*) is an important zoonotic parasite that infects many mammals, including cats, dogs, and humans.* G. lamblia* assemblage A and B infection in individuals can lead to abdominal cramps, acute or chronic diarrhea, and malabsorption [1]. Since the 1970s, the disease has been epidemic or outbreak all over the world; more than 280 million of human symptomatic infections each year only in Africa, Asia, and America are estimated by the World Health Organization (WHO) [2, 3].

*G. lamblia* has two stages of life cycle: an infective, immotile cyst and a vegetative, motile trophozoite. The latter (trophozoite) has a pear-shaped body, which is ventrally flattened and bilaterally symmetric. Besides, it has two adhesive disks on the ventral surface, a microtubular median body, two nuclei, and four pairs of flagella: anterior, ventral, lateroposterior, and caudal [4]. Giardins, unique component of the cytoskeleton of* Giardia*, have been identified as four major classes: alpha (*α*), beta (*β*), gamma (*γ*), and delta (*δ*) [5]. Among them, 21 *α*-giardins numbered from *α*-1 to *α*-19 giardin (*α*-7 giardin subdivided as *α*-7.1, *α*-7.2, and *α*-7.3) have been discovered; their relative molecular masses are about 29~38 kDa, with common characteristic of calcium dependency membrane bound proteins containing acidic phospholipid. They are often associated with the plasma membrane and membrane systems and participate in the movement of the cytoskeleton and signal transduction in the cell, as well as regulating the growth and proliferation of cells and forming the atypical Ca^2+^ channels [6, 7]. In addition, some *α*-giardins also participate in the encystation and excystation process of* Giardia* cysts, preventing their leakage of the cell membrane and disintegration of the cellular structure under the environment of high intestinal bile salts [6].

In recent years, numerous researches on the subcellular localization of *α*-giardins have been reported. Weiland et al. [6] identified 14 coding genes for *α*-giardins (*α*-4 to *α*-6, *α*-8 to *α*-13, and *α*-15 to *α*-19) in* G. lamblia*. The localization experiments showed that a few *α*-giardins (*α*-3, *α*-5, and *α*-17) are localized in the adhesive disc; *α*-15 and *α*-16 giardins had a patchy distribution along the plasma membrane, while the others (*α*-1, *α*-2, *α*-6, *α*-7.2, *α*-7.3, *α*-9, *α*-10, and *α*-14) were mainly distributed in plasma membrane, adhesive disc, flagella, or cytoplasm. Alpha 1 (*α*-1) giardin was identified mainly present in plasma membrane in strains of assemblages A but also in assemblages B and E [8]. Alpha 8 (*α*-8) and *α*-4 giardins were found to be mainly located on the flagella and plasma membrane (the former) and flagella by specific polypeptide, respectively [9, 10]. The immunofluorescence assay revealed that *α*-11 giardin is mainly localized in the plasma membrane and basal body of the anterior flagella of* G. lamblia* trophozoite [11]. Recently we have successes on sequence analysis and prokaryotic expression of* G. lambliaα*-18 giardin gene [12]. And we founded that *α*18- and *α*12-giardin proteins were mainly localized in the flagella and cytoplasm of trophozoites, respectively [13]. However, there are no reports about *α*-13 giardin to date.

In this research work, we proceed to provide new reports on prokaryotic expression of *α*-13 giardin gene and its intracellular localization in* G. lamblia* assemblage A to lay a foundation for further functional study of *α*-13 giardin.

## 2. Materials and Methods

### 2.1. Ethics

Animal experiments involved in this study were treated in accordance with the guidelines of the South China Agricultural University Animal Care and Use Committee, which operates under the Animal Welfare Law and Regulations of the Department of Health and Human Services. The South China Agricultural University Animal Care and Use Committee has approved all protocols of this study.

### 2.2. Cell Culture

Trophozoites of* G. lamblia* strain WBC6 (ATCC 50803) were cultured in Keister's modified TYI-S-33 medium [14] at 37°C, which was placed in 10 mL glass tubes. The cultures were decanted into 15 mL centrifuge tubes at the end of the logarithmic phase (after about 3 days), and trophozoites were harvested at 5400 ×g for 10 min at 4°C.

### 2.3. PCR Amplification

Total DNA was extracted from trophozoites using a commercial DNA Extraction Kit (Promega, Madison, USA) and quantified by spectrophotometry at 260 nm. Two primers specific to *α*-13 giardin gene, A13E (5′-CGG GAT CCA TGC CTG TTC TGA CCC C-3′) with* Bam* HI restriction site (underlined) and A13F (5′-CCC AAG CTT CTA ATC CAC ATC CCA GAG CC-3′) with* Hind* III restriction site (underlined), were designed based on the* G. lamblia* nucleotide sequence (GL50803_1076). The predicted amplification fragment was 1038 bp. A 25 *μ*L of PCR reaction mix contained 17.3 *μ*L of ddH_2_O, 2.5 *μ*L of 10x PCR Buffer (Mg^2+^ plus), 2.5 *μ*L dNTP, 0.2 *μ*L of upstream and downstream primers (50 pmol/L), 0.3 *μ*L of ExTaq DNA polymerase, and 2 *μ*L of DNA sample. The thermocycler program consisted of 95°C for 5 min, followed by 35 cycles of 94°C for 30 s, 55.2°C for 30 s, and 72°C for 2 min, and a final extension at 72°C for 7 min. The PCR products were analyzed by electrophoresis in 1% agarose gels, followed by ethidium bromide staining and photographed under UV light.

### 2.4. Construction of Recombinant Plasmids

The obtained PCR products were purified by using DNA gel Extraction Kit (Omega, Guangzhou, China) and cloned into pMD18-T (TaKaRa, Dalian, China) according to the manufacturer's instructions. Then the PMD18-T-g-*α*13 plasmid was transformed into JM109 competent cells. The positive colonies carrying the target gene were selected by fresh LB agar plates supplemented with 100 mg/mL of ampicillin after growing for 12 h at 37°C; then the PMD18-T-g-*α*13 was purified using the Plasmid Mini Kit (Omega, Guangzhou, China). The obtained plasmid and pET-28a (+) were digested with* Bam* HI/*Hind* III (TaKaRa, Dalian, China) and connected by T4 DNA ligase (TaKaRa, Dalian, China) to create recombinant plasmids which were confirmed by PCR and* Bam* HI/*Hind* III digestion and then sequenced by Sangon Biotech Company (Shanghai, China). The correct recombinant prokaryotic expression plasmids were named as pET-28a(+)-g-*α*13.

### 2.5. Expression of *α*-13 Giardin

The recombinant plasmids were transformed into* E. coli* BL21(DE3) (TransGen Biotech, Beijing, China). Freshly transformed bacteria were inoculated into LB medium (50 *μ*g/mL kanamycin) and grew at 37°C with continuous shaking cultivation at 200 rpm until the absorbance at 600 nm reached 0.4~0.6. The *α*-13 giardin fusion protein was expressed under the induction of isopropyl-1-thio-*β*-D-galactopyranoside (IPTG); firstly, the expression conditions were optimized by adjusting IPTG concentrations (0.2, 0.4, 0.6, 0.8, 1.0, and 1.2 mM), induction time (1, 3, 5, and 7 h), and culturing temperature (16, 20, 25, 30, and 37°C). Then, they were expressed by autoinduction expression system (ZYM-5052); the bacterium solutions cultivated by ZYM-5052 for 3~16 h were collected, respectively. For details, refer to Studier [15] with few modifications. The cultures were harvested at 9600 ×g for 10 min at 4°C after washing the cell pellet in phosphate buffer (containing 0.1% Tween-20).

### 2.6. SDS-PAGE and Western Blotting Analysis

Samples containing 40 *μ*L of total proteins were boiled for 10 min after adding 10 *μ*L 5x SDS loading sample buffer, then stored for 5 min at −20°C, and centrifuged at 16200 ×g for 5 min. Then, 10 *μ*L supernatant of each sample was analyzed by SDS-PAGE performed in Tris-glycine system, using 12% gels. Proteins were visualized by staining with Coomassie brilliant blue G-250. The target protein expression was detected by LiCor Odyssey infrared Imaging system (LiCor, Nebraska, USA) and analyzed by Quantity One 1-D Analysis Software (Bio-Rad, Hercules, USA). Ten microliters of each preprocessed protein sample was separated on a 12% SDS-PAGE at 120 V and transferred to nitrocellulose membrane (Roche, Indianapolis, USA) for 45 min at 120 mA. The membrane was blocked overnight at 4°C in 0.1% Tween-20-PBS containing 5% nonfat milk. The *α*-13 giardin proteins were detected using the ECL Plus Western blotting detection system (Tiannon, Shanghai, China) by rabbit anti-His tag monoclonal antibody (diluted 1 : 2000) and horseradish peroxidase- (HRP-) labeled goat anti-rabbit IgG antibody (Transgen, Beijing, China) at dilution of 1 : 3000.

### 2.7. Protein Purification

For purification of soluble *α*-13 giardins, BL21 (DE3) cells harboring pET-28a(+)-g-*α*13 were cultured in 1000 mL of LB medium for 5 h at 25°C with 50 *μ*g/mL kanamycin. The cultivated cells were harvested by centrifugation at 8,000 ×g for 10 min, resuspended in PBS, and subjected to sonication (SCIENTZ, Ningbo, China). Soluble and insoluble fractions were separated by centrifugation at 10000 ×g for 30 min at 4°C. The supernatant was applied on a Ni^2+^ nitrilotriacetic acid column in binding buffer (0.3 M NaCl, 50 mM NaH_2_PO_4_, 10 mM Tris-base, and pH 8.0), eluted with different concentrations of imidazole (0.01, 0.02, 0.05, 0.1, 0.15, and 0.2 M), and analyzed by SDS-PAGE. The total protein concentrations in the supernatant were determined by using a Bicinchoninic Acid (BCA) Protein Assay Kit (ComWin Biotech, Beijing, China) according to the manufacturer's instructions.

### 2.8. Preparation of Anti-*α*13-Giardin Polyclonal Antibody

The anti-*α*13-giardin polyclonal antibody was prepared as described [16] with some modifications. Briefly, two male New Zealand white rabbits (2.5 kg) were inoculated with 1 mL (1 mg/mL) purified anti-*α*13-giardin protein emulsified with an equal amount of Freund's complete adjuvant via subcutaneous injection. Immunization of each rabbit was boosted four times by inoculation of 1 mL antigen mixed with an equal volume of Freund's incomplete adjuvant at 1-week intervals. Blood sample was collected 7 days after final injection; the sera were obtained to determine the antibody titer by indirect enzyme linked immunosorbent assay (ELISA). Prior to first immunization, a certain amount of serum was taken from ear marginal veins of each rabbit as negative control.

### 2.9. Determination of Antibody Titers by ELISA

The antibody titers were determined using indirect ELISA as described [17]. Briefly, purified *α*13-giardin proteins were coated on plates at 200 *μ*L per well in 96-well plates at 4°C overnight. The plates were washed three times with PBS containing 0.1% Tween-20 (PBST). The coated wells were blocked for 1 h at 37°C with 200 *μ*L of 5% nonfat milk in PBST, then washed three times with PBST, and incubated with 100 *μ*L of anti-*α*13-giardin serum by serial dilutions (from 1 : 100 to 1 : 243,000, normal serum as negative control). After incubation for 1 h at 37°C, the wells were washed and incubated with 200 *μ*L of HRP-conjugated goat anti-rabbit IgG antibody (dilution, 1 : 4,000; Transgen, Beijing, China). The reaction was terminated with the stop buffer, and the absorbance at 450 nm was measured using a microplate reader (Allsheng, Hangzhou, China).

### 2.10. Determination of Antisera Specificity

*G. lamblia* trophozoite lysates were extracted using Total Protein Extraction Kit (Vazyme, Nanjing, China) according to manufacturer's instructions. Total trophozoite extract and the purified *α*-13 giardin fusion protein were subjected to 12% SDS-PAGE and transferred to nitrocellulose membrane. The membrane was blocked at 37°C for 1 h with 5% nonfat dry milk in PBST, followed by incubation with the anti-*α*13 giardin antibody (1 : 200 dilution) at 4°C overnight. After three washes with PBST (containing 0.1% Tween-20), the membrane was incubated for 1 h at 37°C with HRP-conjugated goat anti-rabbit IgG (1 : 2000 dilution). Then the membrane was rinsed three times, and the protein bands were visualized with an enhanced chemiluminescence protocol and the Western blotting detection system (Tiannon, Shanghai, China).

### 2.11. Immunofluorescence (IF) Staining of* G. lamblia* Trophozoites

The immunofluorescence staining of* G. lamblia* trophozoites was carried out as described in previous reports [11, 18] with some modifications. Briefly,* G. lamblia* trophozoites were allowed to attach to sterile glass coverslips, fixed for 15 min with methanol, and permeabilized for 10 min at −20°C with 0.5%–1% Trion X-100/PBS. The cells were rinsed three times with PBS for 5 min each time. And they were incubated for 60 min with blocking buffer (5% goat serum) (ComWin Biotech, Beijing, China). Primary antibody was diluted in dilution buffer (at 1 : 500), after reacting with the rabbit anti-*α*13-giardin antiserum for 1 h; the samples were washed three times with PBS and then incubated for 1 h in the dark with Alexa Fluor 488 anti-rabbit IgG (H+L) from goat (1 : 200) (Beyotime, Jiangsu, China) at room temperature. Coverslips were kept in a humidified chamber to avoid evaporation. They were rinsed three times with PBS before counterstaining with DAPI (4′,6′-diamidino-2-phenylindole; Genecopoeia, Rockville, USA). After five washes with PBS, fluorescence staining was visualized by using Nikon DS-Qi2 Imaging fluorescence microscope (Nikon, Tokyo, Japan), with 100x oil immersion objectives (Size: 1608 × 1608), and images were processed using NIS Elements (version 4.50) Imaging software.

## 3. Results

### 3.1. Construction of Recombinant Plasmid

**Figure 1:**
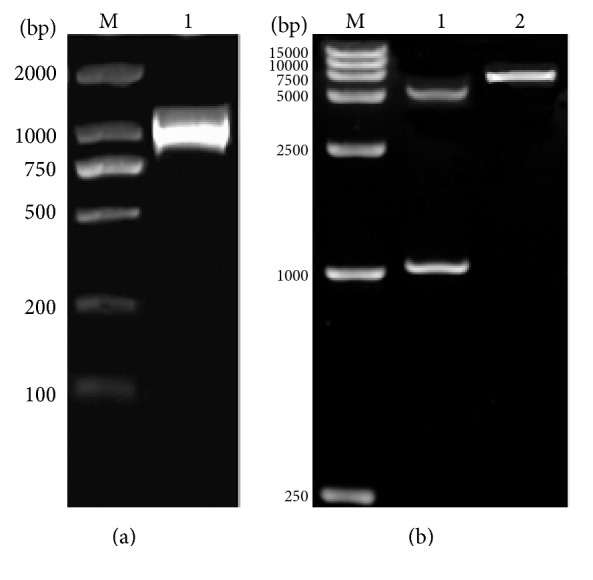
Amplification of *α*-13 giardin gene (a) and identification of recombinant plasmid pET-28a(+)-g-*α*13 (b). (a) M, DNA marker DL-2,000 and 1, PCR amplicons. (b) M, DNA marker DL-15,000; 1, digested pET-28a(+)-g-*α*13; and 2, undigested pET-28a(+)-g-*α*13.

PCR amplification from *α*-13 giardin gene showed that the specific fragment was about 1000 bp, which was consistent to the expected size (Figure 1(a)). The identification of recombinant plasmid pET-28a(+)-g-*α*13 digested with* Bam* HI and* Hind* III was proved to be successful (Figure 1(b)).

### 3.2. The Expression of *α*-13 Giardin after Induction

**Figure 2:**
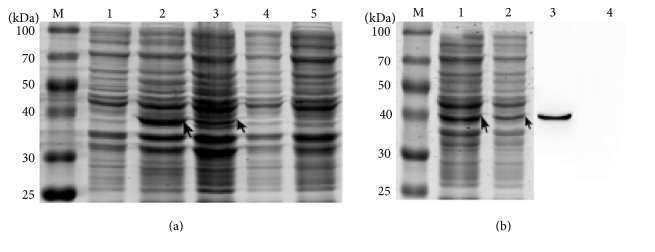
SDS-PAGE (a, b) and Western blot (b) analysis of *α*-13 giardin fusion protein. (a) M, standard protein marker; 1, pET-28a(+)/BL21(DE3); 2, total cell lysate of pET-28a(+)-g-*α*13 induced with ZYM-5052 medium; 3, total cell lysate of pET-28a(+)-g-*α*13 induced with 0.5 mM IPTG; 4, total cell lysate of uninduced pET-28a(+)-g-*α*13; and 5, BL21(DE3). (b) M, standard protein marker; 1, ultrasound supernatant of pET-28a(+)-g-*α*13/BL21(DE3); and 2, ultrasound precipitation of pET-28a(+)-g-*α*13/BL21(DE3). (b) 3, total cell lysate of pET-28a(+)-g-*α*13/BL21(DE3) induced with 0.5 mM IPTG and 4, total cell lysate of pET-28a(+)-g-*α*13/BL21(DE3).

The results showed that the target protein reached the maximum and accounted for 21.0% of the total cell proteins after induction with 0.6 mmol/L IPTG for 5 h at 25°C. Comparing the induction effect by ZYM-5052 with conventional IPTG induction at the same temperature, the results showed that the target protein reached the maximum and accounted for 28.8% of the total cell proteins after being induced by ZYM-5052 for 14 h at 37°C, while it accounted for 6.7% of the total cell proteins after being induced with 0.6 mmol/L IPTG for 5 h at 37°C (Figure 2(a)).

### 3.3. Identification and Purification of *α*-13 Giardin

**Figure 3:**
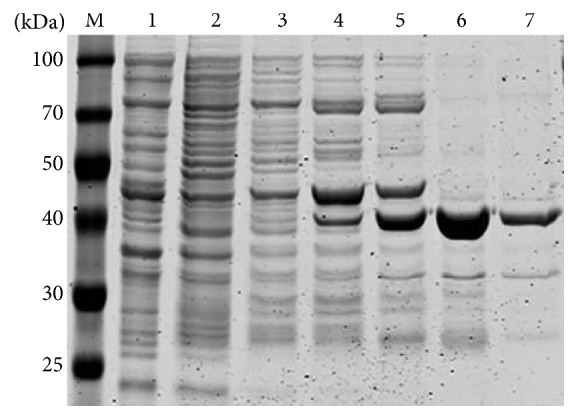
SDS-PAGE analysis of *α*-13 giardin fusion protein after purification by Ni-NTA Resin column. M: standard protein marker; 1: cell lysate (supernatant); and 2–7: proteins eluted with imidazole of 10, 20, 50, 100, 150, and 200 mmol/L.

SDS-PAGE results showed that both the supernatant and precipitate contained the target protein, but the supernatant had a higher amount of the target protein than the precipitate, indicating that the recombinant proteins mainly existed in soluble form (Figure 2(b)). The Western blotting analysis showed that the rabbit anti-His tag monoclonal antibody and HRP-labeled goat anti-rabbit IgG antibody reacted with a protein of about 40 kDa (Figure 2(b)), which is consistent with the theoretical value of the *α*-13 giardin. SDS-PAGE analysis indicated that the *α*-13 giardin fusion protein could be eluted with 0.1, 0.15, and 0.2 mM imidazole (Figure 3). However, the eluate with 0.1 mM imidazole contained several impurity proteins, while the one with 0.2 mM imidazole had loss of the target protein, so the eluate with 0.15 mM imidazole was more satisfactory. The protein detecting results revealed that the total protein concentration was 1.17 mg/mL.

### 3.4. Specificity of Anti-*α*13 Giardin Polyclonal Antibody

**Figure 4:**
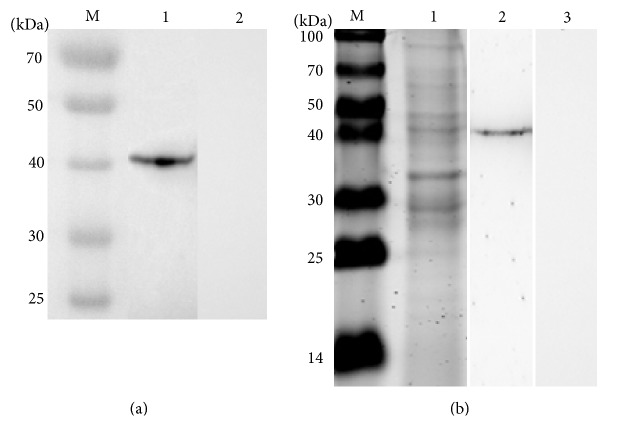
Confirmation of specificity of anti-*α*13 giardin antibodies. (a) Western blotting of recombinant *α*-13 giardin proteins. 1, reactivity of the anti-*α*13-giardin antibodies with the recombinant proteins and 2, control with preimmune serum. (b) Western blotting of* G. lamblia* trophozoite lysates separated by SDS-PAGE (12% acrylamide). M, standard protein marker; 1, Coomassie stain of total trophozoite lysates; 2, specific reactivity of the anti-*α*13 giardin antibodies with trophozoite lysates; and 3, control with preimmune serum.

The anti-*α*13 giardin polyclonal antibody titer detected by ELISA was more than 1 : 243,000. The candidate antisera reacted with recombinant *α*-13 giardin expressed in* E. coli* as a polyhistidine fusion protein (Figure 4(a)) and were specific to a 40 kDa protein band with total trophozoite lysates in Western blot assay (Figure 4(b)). Results showed that the polyclonal antibody was specific to the *α*-13 giardin of trophozoites.

### 3.5. Intracellular Location of *α*-13 Giardin in Trophozoites

**Figure 5:**
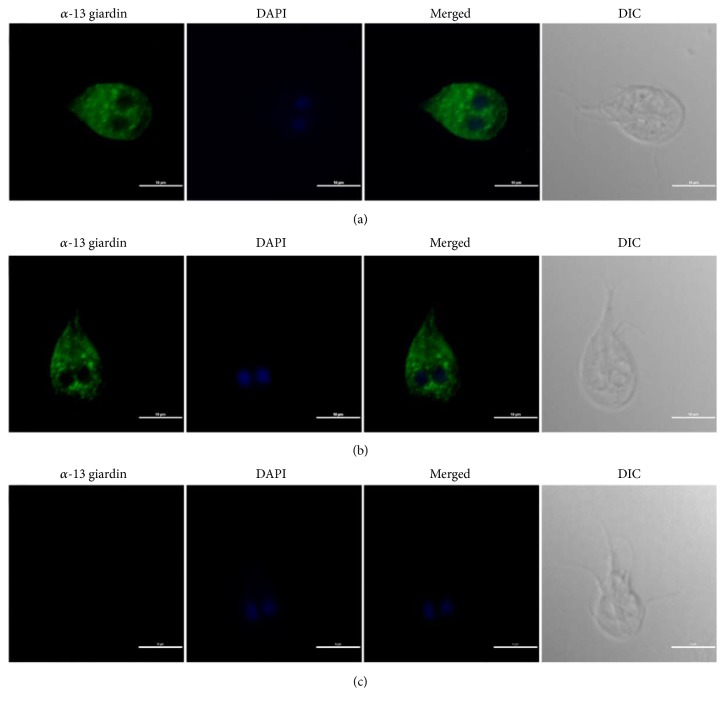
Immunofluorescent localization of *α*-13 giardin in* G. lamblia* trophozoites. (a, b): Fluorescence microscopy images of single trophozoite (green) incubated with anti-*α*13-giardin antiserum. (c): Control with preimmune serum. DIC, bright-field differential interference contrast. Nuclear DNA is stained blue with DAPI. The scale bar is 10 *μ*m.

Immunofluorescence assay showed that the fluorescence signals of Alexa Fluor 488 anti-rabbit IgG (H+L) from goat were significantly distributed in the cytoplasm of trophozoite but not in the flagella and plasma membranes (Figures 5(a) and 5(b)). The preimmune serum (control) did not react with the trophozoite, and no fluorescence signal appeared (Figure 5(c)). It revealed that *α*-13 giardin was localized in the cytoplasm in trophozoites of* G. lamblia* assemblage A.

## 4. Discussion

Four classes of giardins, *α*, *β*, *γ*, and *δ*, have been identified as cytoskeleton components of the* Giardia*. The genome of* G*.* lamblia* possesses a total of 21 *α*-giardin encoding genes, which are *α*-giardins numbered from *α*-1 to *α*-19 giardin (*α*-7 giardin subdivided as *α*-7.1, *α*-7.2, and *α*-7.3). The different *α*-giardins have great differences in the protein expression level, the subcellular localization, and biological functions. The overexpression of some *α*-giardins often affects the division, proliferation, and differentiation of the cells, even leading to death of the parasite, which suggests that the *α*-giardins possess important biological functions.

This work presents the first report on prokaryotic expression of the *α*-13 giardin gene of* G. lamblia*. There are many factors that affect the expression of foreign genes, such as, the target encoding gene, the expression vector, the competent cell, the cultivation condition of the* E. coli*, and the induction methods [19]. Previous studies [6, 19, 20] have indicated that the genome of the eukaryotic protist* Giardia* is compact in structure, contains few introns, and has simplified machinery for DNA replication and transcription. And *α*-giardin genes have been known to lack introns, so *α*-13 giardin gene was cloned directly using genomic DNAs in the present study. The results showed that the amplification of *α*-13 giardin gene, construction of pET-28a(+)-g-*α*13, and the expression of *α*-13 giardin in BL21(DE3) were successful. However, in the initial stage of experiment, the production of target protein was very low and mainly existed in a form of inclusion body at 37°C as the optimal temperature for bacterial growth. Moreover, some expressing host cells had lost their expression plasmid. To overcome these limitations, we tried two solutions. Firstly, we compared the expression and solubility of fusion protein at 37°C, 30°C, 25°C, 20°C, and 16°C with different IPTG concentrations (0.2, 0.4, 0.6, 0.8, 1.0, and 1.2 mM) and different induction time (1, 3, 5, 7, 9, and 11 h); the optimized induction condition was for 5 h at 25°C with 0.5 mM IPTG to the recombinant BL21 at OD_600_ 0.8. Secondly, they were expressed by autoinduction expression system (ZYM-5052); the results show that the quantity and stability of the functional recombinant plasmid in the autoinduction expression system had been improved. This work demonstrates the importance of selection of heterologous host and induction methods for successful production of soluble recombinant protein.

Different giardins have different subcellular localizations in* G. lamblia* trophozoites and may play different biological functions. Many *α*-giardins (*α*-2, *α*-4, *α*-5, *α*-8, *α*-9, *α*-10, *α*-11, *α*-14, *α*-17, and *α*-19) have been shown to localize in flagella; a few *α*-giardins such as *α*-3, *α*-5, and *α*-17 were found localized in the disc; some *α*-giardins such as *α*-1, *α*-2, *α*-7.2, *α*-7.3, *α*-8, and *α*-11 were located in the plasma membrane, and other *α*-giardins (*α*-6, *α*-7.3, *α*-12, *α*-15, and *α*-16) had spotty cytoplasmic distribution [6, 9–11, 13, 18]. Previous studies [6, 7] indicated that *α*-giardins are mainly involved in the cytoskeleton, flagella motility, membrane stability, and attachment of* Giardia*. Recent studies [21–23] revealed that *α*-1 giardin contains an epitope between amino acids 160 and 200 that is highly immunogenic and can stimulate the production of anti-*Giardia* antibodies (IgA and IgG2a), which is a suitable candidate antigen for a vaccine against giardiasis. In addition, compared with* Giardia* albendazole- (Abz-) sensitive clones, Abz-resistant clones had upregulated the *α*-2 giardin, which suggests that *α*-2 giardin may function as annexin to cope with reactive oxygen species in Abz-resistant clones [24]. These discoveries of the functions of *α*-giardins will aid in further research on the prevention and control of giardiasis. However, there are no reports about the localization and relevant biological functions of *α*-13 giardin to date. The present study on *α*-13 giardin localization extended the list of giardins located in the cytoplasm. Though the precise function of *α*-13 giardin remains unknown, its specific localization in the cytoplasm suggests that the protein may be associated with a particular cytoplasmic structure like free ribosomes or profiles of the endoplasmic reticulum and it may make contact with the cytoskeleton within the parasite. The exact subcellular location and specific biological function of *α*-13 giardin need further research in the future.

In conclusion, this work presents the first report on prokaryotic expression of the *α*-13 giardin gene and its subcellular location of* G. lamblia* assemblage A (strain WBC6). It may lay the foundation for further functional research on *α*-13 giardin of zoonotic* G. lamblia* assemblage A.
